# Characterization and anti-tumor activity of a polysaccharide isolated from *Dendrobium officinale* grown in the Huoshan County

**DOI:** 10.1186/s13020-018-0205-x

**Published:** 2018-09-10

**Authors:** Yuan Wei, Linwei Wang, Dujun Wang, Dan Wang, Chongwei Wen, Bangxin Han, Zhen Ouyang

**Affiliations:** 1 School of Pharmacy, Jiangsu University, 301 Xuefu Road, Zhenjiang, 212013 Jiangsu China; 2 School of Biological and Pharmaceutical Engineering, West Anhui University, Lu’an, 237012 China

**Keywords:** *Dendrobium officinale*, Huoshan area, Polysaccharide characterization, Anti-tumor effect, Apoptosis

## Abstract

**Background:**

Polysaccharides are carbohydrate chains composed of linked monosaccharide units. Accumulating studies report that polysaccharides isolated from *Dendrobium officinale* have a variety of functions. However, the composition and anti-tumor activity of *D. officinale* grown in the Huoshan area are largely unknown.

**Methods:**

A polysaccharide (DOPA-1) was isolated from *D. officinale* by hot water extraction and ethanol precipitation, followed by purification via DEAE-cellulose and Sephadex G-100 chromatography. DOPA-1 was analyzed by infrared and nuclear magnetic resonance and then characterized by periodate oxidation and Smith degradation. The anti-tumor activity of DOPA-1 was then tested in HepG-2 cells.

**Results:**

Our results show that DOPA-1 is mainly comprised of mannose, glucose, and galactose at a molar ratio of 1:0.42:0.27 and has an average molecular weight of 2.29 × 10^5^ Da. Additionally, DOPA-1 inhibited HepG-2 cell growth in a dose-dependent manner. DOPA-1-treated HepG-2 cells also had increased reactive oxygen species (ROS) levels and decreased mitochondrial membrane potential. Furthermore, apoptosis was observed in DOPA-1-treated HepG-2 cells along with Bcl-2 downregulation and Bax upregulation at the protein level.

**Conclusions:**

Our findings suggest that DOPA-1 induces apoptosis in tumor cells via altered mitochondrial function, ROS production, and altered apoptosis-related protein expression. This bioactive polysaccharide could, therefore, potentially be further developed as an anti-tumor adjuvant drug.

## Background

*Dendrobium officinale* Kimura et Migo is a rare and endangered perennial orchid found in South and Southeast Asia. It is valued for its medicinal uses, including the treatment of fatigue, night sweats, fever, infantile convulsion, palpitations, and dizziness, and has been utilized in traditional Chinese medicine for more than 1000 years [1]. *D. officinale* grown in the Huoshan area, Anhui province of China, is recognized by its ability to grow in extremely harsh conditions. It is also considered to be the top grade of *D. officinale* by many researchers [2].

Polysaccharides are one of the main bioactive substances of many fungi, algae, and higher plants [3]. They are polymeric carbohydrate molecules composed of long chains of monosaccharide units. Polysaccharides play manifold roles and have broad activities in biochemical reaction, with an immense potential in healthcare and food industries, due to their therapeutic effects [4]. Accumulating evidence has shown that polysaccharides isolated from natural plants are nontoxic and have a variety of functions including inhibition of tumor cell proliferation [5], induction of tumor cell apoptosis [6], enhancement of immunity [7], and cellular protection from oxidative stress [8]. In addition, polysaccharides have also been found to reduce multidrug resistance and could potentially be applied as adjuvant drugs for chemotherapy [9].

In recent years, special attention has been paid to polysaccharides from *D. officinale* [10], as they have been reported to have significant anti-tumor [11], anti-oxidative [12], immunomodulatory [13], and blood sugar reducing [14] effects. Furthermore, the combination of *D. officinale* polysaccharides and recombinant interleukin (IL)-2 appears to be particularly effective at significantly enhancing PB-LAK cytotoxicity [15]. However, while polysaccharide content and activity have been evaluated in *D. officinale* samples from various origins, those of *D. officinale* from the Huoshan area have yet to be fully characterized, particularly with regards to their composition and anti-tumor activity.

In the present study, we isolated and purified a unique polysaccharide sample from *D. officinale* from the Huoshan are (designated DOPA-1) and then characterized its structure as well as its anti-tumor activities in HepG-2 cells. To the best of our knowledge, this is the first time this promising bioactive polysaccharide has been evaluated with regards to its composition and anti-tumor mechanisms.

## Methods

### Chemicals and reagents

*Dendrobium officinale* samples grown in the Huoshan area were provided by Tian-Xia-Ze-Yu Biological Technology Development Co., Ltd. (Huoshan County, Anhui, China). These samples were verified by Prof. Zhen Ouyang prior to being cut into small pieces no more than 3 mm in size.

Fetal bovine serum (FBS) and Dulbecco’s modified Eagle’s medium (DMEM) were obtained from Gibco (Grand Island, NY, USA). Standard monosaccharides (d-glucose, d-xylose, d-galactose, l-rhamnose, d-mannose, and d-arabinose), DEAE-52 cellulose, Sephadex G-100, dimethyl sulfoxide (DMSO), 3-(4,5-dimethylthiazol-2-yl)-2,5-diphenyltetrazolium bromide (MTT), 5-fluorouracil (5-FU), propidium iodide (PI), trypsin, JC-1, and the Annexin V-FITC/PI apoptosis detection kit were obtained from Sigma Aldrich (St. Louis, MO, USA). The Bax, Bcl-2, and β-actin antibodies were obtained from Santa Cruz Biotechnology (Santa Cruz, CA, USA). The DCFH-DA reactive oxygen species (ROS) assay and LDH cytotoxicity assay kits were purchased from Beyotime Institute of Biotechnology (Jiangsu, China). HepG-2 cells were provided by the Cell Bank of the Chinese Academy of Sciences (Shanghai, China). All other chemical reagents were of analytical grade. The Minimum Standards of Reporting Checklist contains details of the experimental design, and statistics, and resources used in this study.

### Extraction and isolation

The components of *D. officinale* (20 g) were extracted three times with distilled water (400 mL × 3) for 2 h at 90 °C. The filtrate was combined and concentrated to 20 mL using a rotary evaporator at 60 °C. The concentrated supernatant was then evaporated under reduced pressure and precipitated by adding 95% ethanol until the total ethanol concentration reached 85%. The samples were subsequently stored at 4 °C overnight. The precipitate was collected following centrifugation (4000 rpm, 15 min), dissolved in distilled water, and lyophilized to obtain the crude polysaccharides. The crude polysaccharides were then deproteinized using the enzyme-Sevag method [16]. After the crude polysaccharides were dissolved in distilled water and fractionated on DEAE-52 cellulose, they were successively eluted with distilled water and an NaCl gradient (0.1–0.3 M) at 1.0 mL/min. The carbohydrate content of each fraction (10 mL/each) was monitored at 490 nm using the phenol–sulfuric acid method [17]. The obtained eluates were then combined, concentrated, dialyzed, lyophilized, and subjected to further purification on a Sephadex G-100 column. The fractions were subsequently combined and lyophilized to obtain a purified polysaccharide sample. The major fraction with only one main peak was lyophilized and named DOPA-1.

### Homogeneity, molecular weight, and monosaccharide composition analysis

The homogeneity and molecular weight of DOPA-1 was determined with high performance liquid chromatography (HPLC) using a TSK-GEL G-4000 PWXL column (7.8 × 300 mm, column temperature 30 °C) and a Waters alliance refractive index detector (RID, detection temperature 30 °C). Prior to injection, the sample was filtered through a 0.45 μm membrane. Then, 10 μL of DOPA-1 (1.0 mg/mL) was injected into the HPLC column and eluted with distilled water at a flow rate of 0.45 mL/min. Dextrans of varying molecular weight (10, 40, 70, 500, and 2000 kDa) were used to establish a standard curve.

The monosaccharide composition of the purified DOPA-1 was measured by gas chromatography (GC). Briefly, 10 mg of the polysaccharides were hydrolyzed in a sealed glass tube with 5 mL of 2 M sulfuric acid at 100 °C for 8 h. The acid was then neutralized with barium carbonate. Next, the hydrolysate was acetylated with 10 mg of hydroxyl-amine hydrochloride in 1 mL of pyridine for 30 min at 90 °C. After cooling to 20–25 °C, 1 mL of acetic anhydride was added and further incubated for 30 min at 90 °C. Following cooling, the corresponding aldononitrile acetate derivatives were obtained and the corresponding monosaccharides (rhamnose, arabinose, xylose, mannose, glucose, and galactose) were then analyzed.

### Infrared spectrum analysis

DOPA-1 (1.0 mg) was ground with KBr (100 mg) and pressed into a pellet. The Fourier transform infrared (FT-IR) spectra were recorded with an FT-IR spectrometer (Nicolet Nexus 470 FT-IR, USA) at a frequency range of 4000–500 cm^−1^.

### Nuclear magnetic resonance (NMR) spectroscopy

DOPA-1 (30 mg) was dissolved in 99.9% deuterium oxide (0.5 mL). The sample was then freeze-dried three times. The ^1^H NMR spectra was recorded with a Bruker DRX-400 NMR spectrometer (Bruker, Rheinstetten, Germany) at 25 °C. Data processing was performed using standard Bruker XWIN-NMR software.

### Periodate oxidation and Smith degradation

DOPA-1 (25 mg) was oxidized in 0.030 M NaIO_4_ (25 mL) in the dark for 120 h and the absorption was monitored every 12 h with a UV spectrophotometer at 223 nm until a constant absorption value was achieved. NaIO_4_ consumption was quantitatively measured using the UV spectrophotometric method [18] and the yield of formic acid was titrated with 0.01 M NaOH. Ethylene glycol (2 mL) was added to remove excess periodate. The periodate product was dialyzed and reduced with NaBH_4_ (40 mg) for 24 h at room temperature. The product was then neutralized to pH 6.0–7.0 with acetic acid. Following dialysis and lyophilization, the product was finally hydrolyzed with 2 M trifluoroacetic acid (3 mL) at 110 °C for 6 h and the hydrolysate was subjected to GC analysis.

### Cell culture and treatment

HepG-2 cells were cultured in DMEM medium supplemented with 10% (v/v) heat-inactivated FBS, 100 U/mL penicillin, and 100 μg/mL streptomycin in a humidified incubator at 37 °C with 5% CO_2_. The medium was refreshed 2–3 times/week.

### MTT cytotoxicity assay

A colorimetric MTT assay was performed to assess cell viability as previously described [19]. Briefly, HepG-2 cells were seeded in a 96-well plate at a concentration of 2.0 × 10^4^ cells/well in 100 μL of medium. Following incubation at 37 °C for 24 h to allow adherence, the cells were treated with different concentrations (50, 100, 200, and 400 μg/mL) of DOPA-1 for 24 h. 5-FU (50 μg/mL) was used as a positive control, while untreated cells were used as a negative control. Following treatment, the medium in each well was removed and 100 μL of MTT (1 mg/mL) was added and incubated at 37 °C for 4 h. Next, the supernatant was aspirated and the crystal violet generated by viable cells was dissolved with 100 μL of DMSO. The absorbance was measured at 570 nm using a microplate reader.

### LDH assay

Cell injury was quantitatively assessed by the measurement of lactate dehydrogenase (LDH). Cells were seeded in 96-well plates. After treatments, a LDH cytotoxicity assay kit (Beyotime, Jiangsu, China) was used to measure the released LDH. Briefly, 100 μl of culture medium was collected from each well. The absorbance of the medium was measured at 490 nm wavelength in an automatic microplate reader. Blank absorbance was subtracted from insult LDH values.

### Determination of ROS levels

A DCFH-DA probe is the most common and sensitive tool for detecting intracellular ROS levels [20]. Logarithmic growth phase HepG-2 cells were digested, counted, and seeded in a 96-well culture plate at a density of 4 × 10^4^ cells/well in 100 μL of medium. After the cells were treated with DOPA-1 for 24 h, the cells were washed twice with phosphate buffered saline (PBS) and incubated with 10 μM of DCFH-DA diluted in serum-free medium at 37 °C for 30 min. The fluorescence intensity was detected with a fluorescence microplate reader at an excitation wavelength of 488 nm and emission wavelength of 525 nm.

### Determination of mitochondrial membrane potential (MMP)

JC-1 is a well-known fluorescent dye used to detect MMP. Following cell treatment, the culture medium was removed and the JC-1 dye (2.5 μg/mL) was added. After incubation at 37 °C for 10 min in the dark, the cells were washed twice with PBS. The fluorescence intensity was detected with a fluorescence microplate reader at an excitation wavelength of 488 nm and emission wavelength of 525/590 nm. The changes in MMP were then observed by fluorescence microscopy.

### Flow cytometry

The rate of apoptosis was determined by cellular staining with Annexin V-FITC and PI [21]. Briefly, the treated cells (4 × 10^5^ cells/mL) were washed twice with ice cold PBS and resuspended in 100 μL of 1× binding buffer. Next, 5 µL of Annexin V-FITC and 5 µL of PI were added and incubated for 10 min in the dark. The cells were then analyzed with a flow cytometer (Guava easyCyte, Merck Millipore, USA).

### Western blotting analysis

Following treatment with DOPA-1 (50, 100, 200, and 400 μg/mL) for 24 h, HepG-2 cells were washed twice with ice-cold PBS and centrifuged at 12,000*g* for 10 min at 4 °C. After adding lysis buffer, the cells were incubated on ice for 20 min. The protein concentration of the supernatant was determined with a BCA protein assay kit. Proteins were separated by sodium dodecyl sulfate (SDS) polyacrylamide gel electrophoresis and then transferred onto a 0.45-μm polyvinylidene fluoride (PVDF) membrane. Subsequently, the membrane was blocked in blocking buffer (0.3 g bovine serum albumin [BSA], 20 mL PBS + Tween 20 [PBST], and 5% non-fat milk) for 2 h at room temperature, followed by incubation with the Bcl-2 (1:1000), Bax (1:1000), or β-actin (1:2000) primary antibodies overnight at 4 °C. The membranes were then washed three times with PBST (15 min each) and incubated with the appropriate secondary antibody (1:2000) for 2 h. Finally, the proteins were visualized with the ECL detection reagent and analyzed using a Chemi Doc XRS imaging system (Bio-Rad, CA, USA).

### Statistical analysis

All experiments were performed in triplicate and the data were expressed as the mean ± standard deviation (SD). All statistical analyses were performed using GraphPad Prism 5. Analysis of variance (ANOVA) was used to compare the groups; p < 0.05 was considered a statistically significant difference.

## Results

### Isolation and purification of DOPA-1

**Fig. 1:**
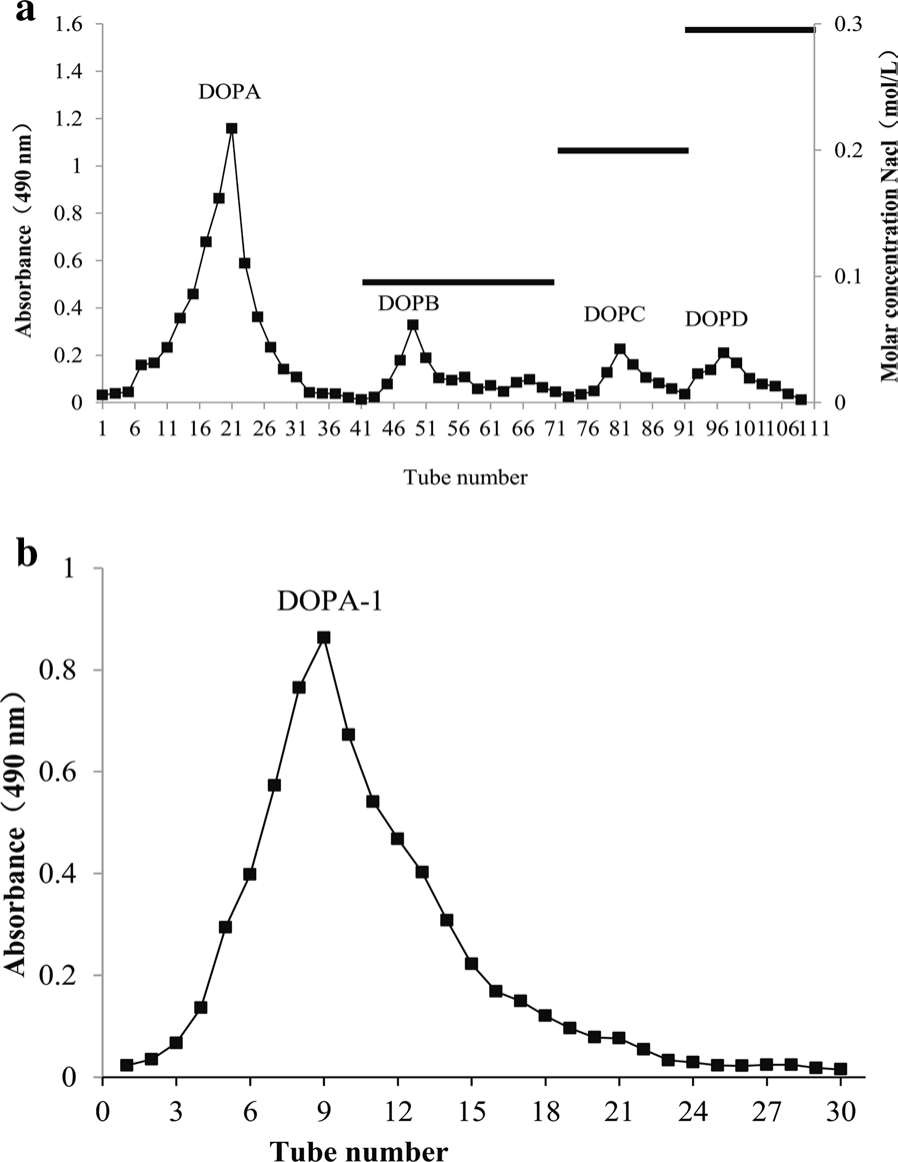
Polysaccharide isolation and purification. **a** Elution profiles of the crude polysaccharide isolated from *D. officinale* grown in the Huoshan area using a DEAE-52 column. **b** Gel filtration chromatography was performed with a Sephadex G-100 column

The polysaccharide extracts from *D. officinale* were obtained by hot water extraction, 95% ethanol precipitation, and lyophilization resulting in an off-white floccule with a yield of 4.25%. The crude polysaccharides were deproteinized, eluted with distilled water, and then successively separated on DEAE cellulose-52 and Sephadex G-100 columns (Fig. 1). The main fraction eluted with distilled water (DOPA-1) was collected for further structural analysis.

### Homogeneity, molecular weight, and monosaccharide composition analysis

**Fig. 2:**
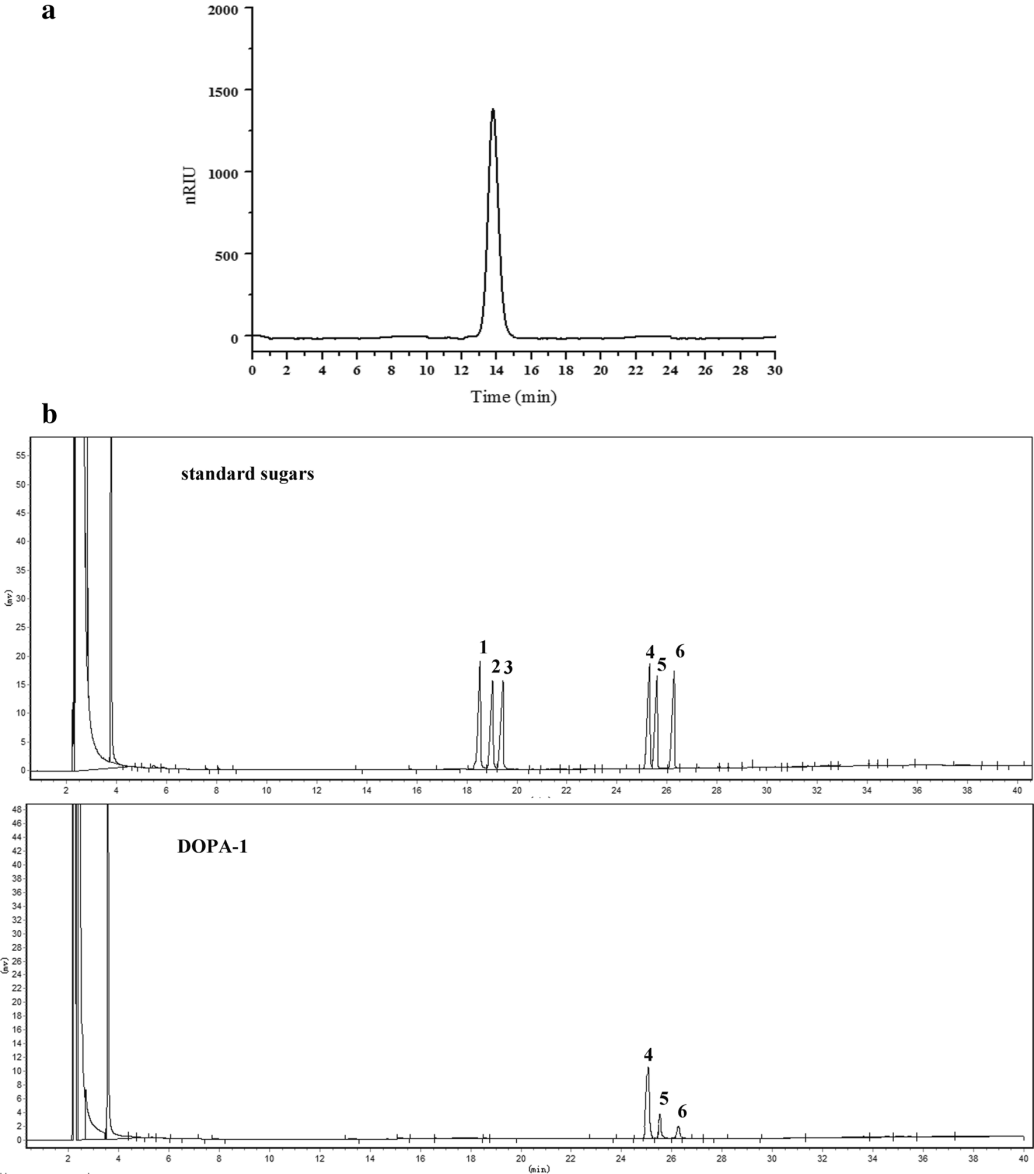
Polysaccharide homogeneity, molecular weight, and monosaccharide composition analysis. **a** HPLC chromatogram of DOPA-1. **b** Monosaccharide composition GC analysis showing the peaks for (1) rhamnose, (2) arabinose, (3) xylose, (4) mannose, (5) glucose, and (6) galactose

The homogeneity, molecular weight, and monosaccharide composition of the purified polysaccharides were determined using HPLC and GC. DOPA-1 showed a single and symmetrically sharp peak at a retention time of 13.789 min (Fig. 2a), illustrating the homogenous nature of this polysaccharide. The average molecular weight of DOPA-1 was determined to be 2.29 × 10^5^ Da based on the calibration data established with standard dextrans. The GC profiles show that DOPA-1 mainly consists of mannose, glucose, and galactose at a molar ratio of 1:0.42:0.27 (Fig. 2b).

### Structural characterization of DOPA-1

**Fig. 3:**
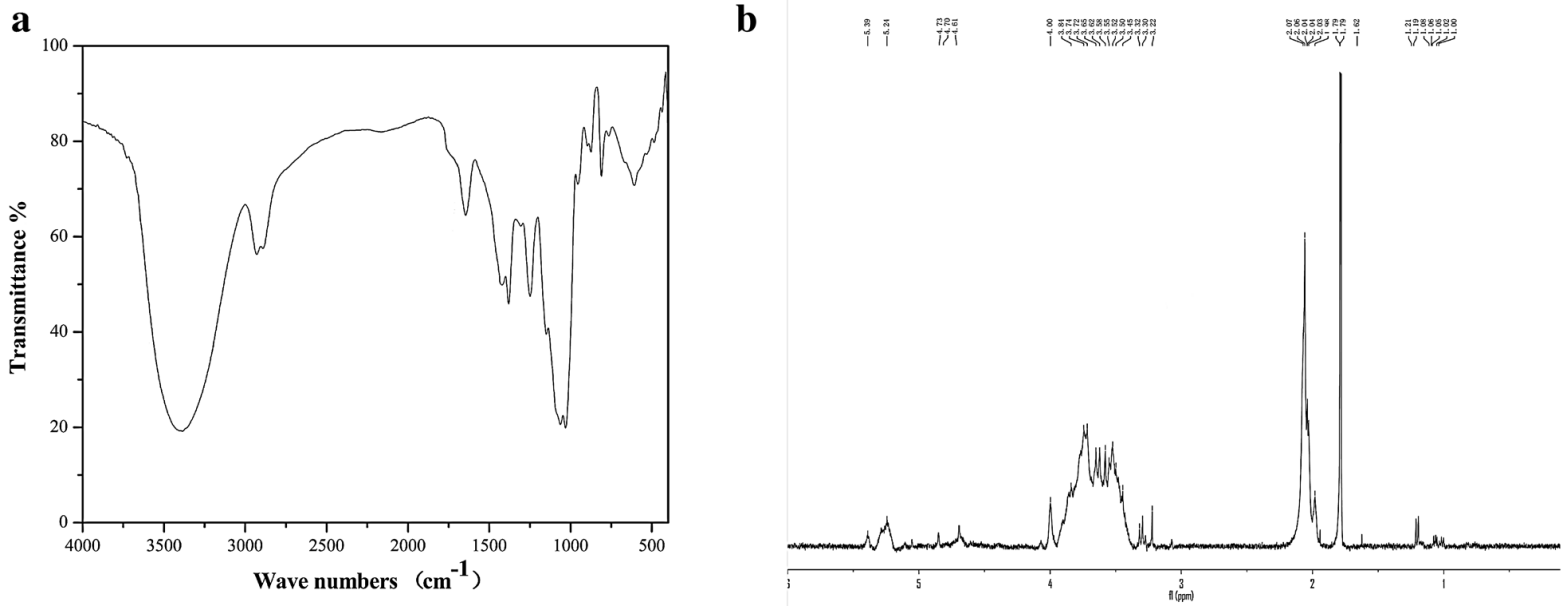
Structural characterization of DOPA-1. **a** FT-IR and **b** ^1^H NMR spectrum of DOPA-1

The FT-IR spectra of DOPA-1 (Fig. 3a) exhibits the characteristic broad and intense absorption peaks at 3386 and 1027 cm^−1^ that are due to the stretching vibrations and deformation vibrations of the hydroxyl group, respectively. The signal at 2929 cm^−1^ has been attributed to the CH stretching vibration, while the signal at 1425 cm^−1^ is distinctive of a CH deformation vibration. The characteristic absorption of C=O (–NHCOCH3) was observed at 1645 cm^−1^. The small band at 1380 cm^−1^ has been attributed to the presence of C=O (–COOH) stretching and the absorptions observed between 1300 and 1000 cm^−1^ indicate the presence of pyranose rings.

In an ^1^H NMR spectrum, the anomeric proton signals of a sugar moiety are based on its conformation, α or β. Most of the signals attributed to α-anomeric protons usually resonate between 5 and 6 ppm, while the signals belonging to the β-anomeric protons resonate between 3 and 5 ppm. Thus, the signal at 5.24 ppm in the ^1^H NMR spectrum of DOPA-1 indicates the presence of a pyranose residue containing an anomeric proton in the α-configuration (Fig. 3b). In addition, the signals resonating between 3.22 and 4 ppm indicate the existence of a β-type glycosidic linkage in DOPA-1.

### Periodate oxidation and Smith degradation

**Fig. 4:**
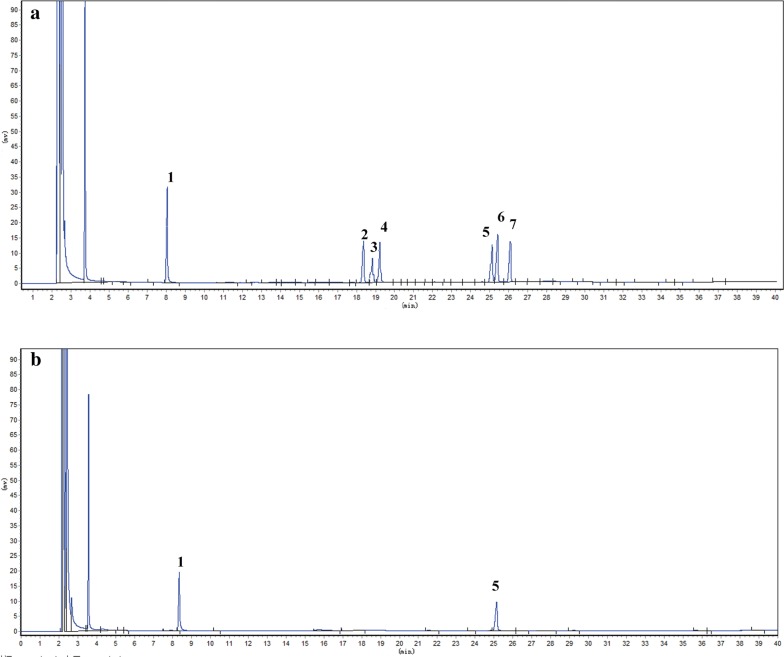
GC chromatogram of DOPA-1 products following Smith degradation. The chromatograms show the peaks for **a** standard sugars and **b** DOPA-1. This includes peaks for (1) rhamnose, (2) arabinose, (3) xylose, (4) mannose, (5) glucose, and (6) galactose

The results of the periodate oxidation reaction show that 0.065 mmol NaIO_4_ was consumed and 0.004 mmol formic acid was generated from DOPA-1, suggesting that an average of 1 mol of hexose residues (the molecular weight of each hexose residue multiplied by 180) consumed 0.389 mol periodate while generating 0.024 mol formic acid. The molar ratio of the hexose residues, sodium periodate consumption, and formic acid generation was 1:0.389:0.024. Thus, it can be inferred that the 1 → 6 linkage residues or the non-reducing end groups of the (1→) linkage in DOPA-1 account for approximately 2.4%, while 34.1% of the residues are likely connected with 1 → 2 or 1 → 4 linkages. Approximately 63.5% of the residues are linked by 1 → 3 linkages. Furthermore, GC analysis of the Smith degradation reaction products detected glycerol and mannose at a molar ratio of 1.32:1. The presence of undegraded mannose indicates that DOPA-1 is composed primarily of 1 → 3 linkages, while glycerol detection implies the presence of 1 → 2 or 1 → 6 linkages in DOPA-1. As no erythritol was found, it can be deduced that there are no 1 → 4 linkages. Taken together, the results of these analyses highlight the existence of 1 → 3, 1 → 2, and 1 → 6 linkages in the main or branched chains of DOPA-1 (Fig. 4).

### DOPA-1 cytotoxicity in HepG-2 cells

**Fig. 5:**
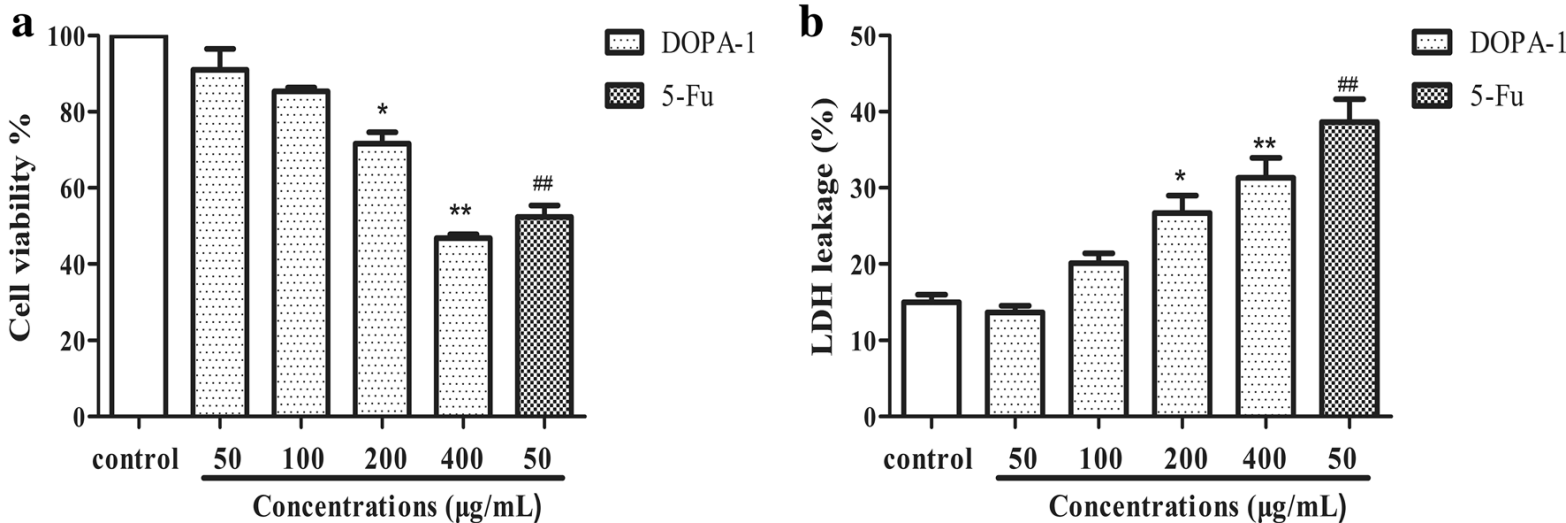
DOPA-1 decreases HepG-2 cell viability. **a** Cell viability was assessed by MTT assay. **b** LDH release assay. The cells were treated with different concentrations of DOPA-1 for 24 h. Data are presented as the mean ± SD of three independent experiments. *p < 0.05, **p < 0.01, ^##^*p *< 0.01, compared to the control

In this study, the effect of increasing concentrations of DOPA-1 (50, 100, 200, and 400 μg/mL) on HepG-2 cell growth was determined using an MTT assay. Notably, DOPA-1 cytotoxicity increased significantly (p < 0.05) in a dose-dependent manner (Fig. 5a). In fact, cell viability following treatment with 50, 100, 200, and 400 μg/mL DOPA-1 for 24 h was 94.51, 85.37, 71.63, and 46.76%, respectively, compared to the 5-FU-treated positive control group (52.4%). The LDH release level increased significantly (p < 0.05) in a dose-dependent manner (Fig. 5b), with the release viability was 13.67, 20.10, 26.67, and 31.32%, respectively, compared to the 5-FU-treated positive control group (38.65%). The effects of DOPA-1 toxicity were approximately consistent between the two assays. Those results indicated that DOPA-1 inhibits HepG-2 cell growth in vitro.

### Effects of DOPA-1 on ROS production

**Fig. 6:**
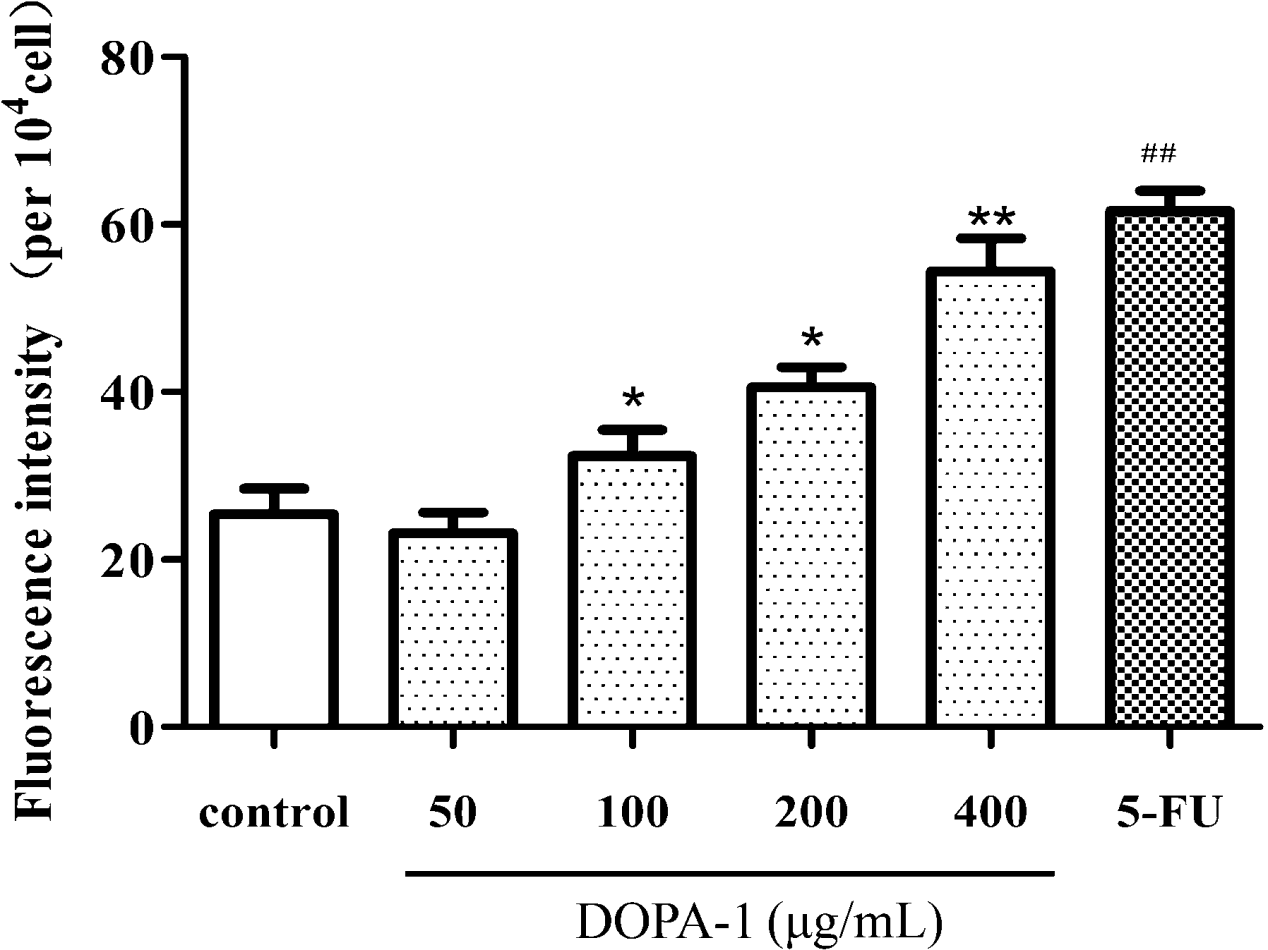
DOPA-1 treatment increases intracellular ROS production in HepG-2 cells. Data are presented as the mean ± SD of three independent experiments. *p < 0.05, **p < 0.01, ^##^*p *< 0.01, compared to the control

The production of excess intracellular ROS can induce apoptosis. Thus, ROS generation in DOPA-1-treated HepG-2 cells was detected by DCFH-DA staining. As highlighted by the increasing fluorescence intensity in Fig. 6, ROS content increased with increasing DOPA-1 concentration in the treated cells. The ROS levels were significantly higher (for all but the lowest DOPA-1 concentration) than those in the untreated control cells. These results suggest that DOPA-1 induces apoptosis in HepG-2 cells via increased intracellular oxidative stress.

### Effects of DOPA-1 on mitochondrial function in HepG-2 cells

**Fig. 7:**
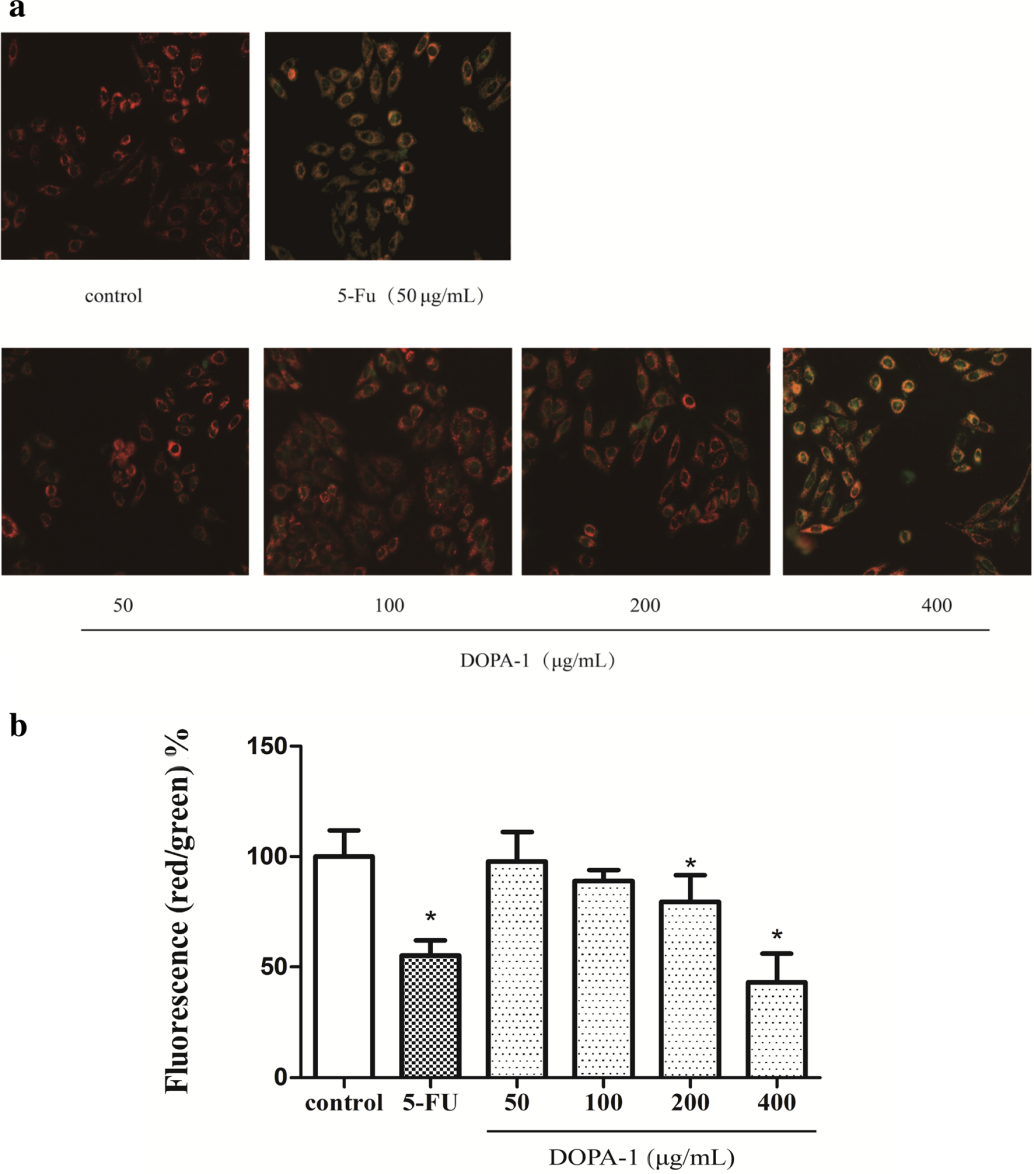
Treatment with DOPA-1 alters mitochondrial function in HepG-2 cells. **a** Fluorescence micrographs of HepG-2 cells stained with JC-1. Changes in cell morphology were observed under ×200 magnification. **b** Quantitative analysis of MMP in HepG-2 cells. Data are expressed as the fluorescence ratio of red to green and are presented as the mean ± SD of three independent experiments. *p < 0.05, compared to the control

Many studies have shown that mitochondria play an important role in apoptosis as well as ROS generation and that changes in MMP can be observed early in the apoptotic signaling cascade [22]. In the untreated controls cells, the fluorescence enzyme marker was mostly red, while green fluorescence was weak, indicating that the mitochondrial membrane was intact and MMP was relatively high (Fig. 7). However, the green fluorescence gradually increased, while the red fluorescence gradually weakened when treated with increasing DOPA-1 concentrations. This shift in fluorescent color signifies increased mitochondrial membrane permeability and decreased MMP as DOPA-1 concentration increased. Indeed, the mitochondrial membrane appeared to be almost entirely disrupted at the highest DOPA-1 concentration. The results of this analysis suggest that DOPA-1-induced apoptosis and increased ROS production are likely related to mitochondrial dysfunction.

### Detection of apoptosis by flow cytometry

**Fig. 8:**
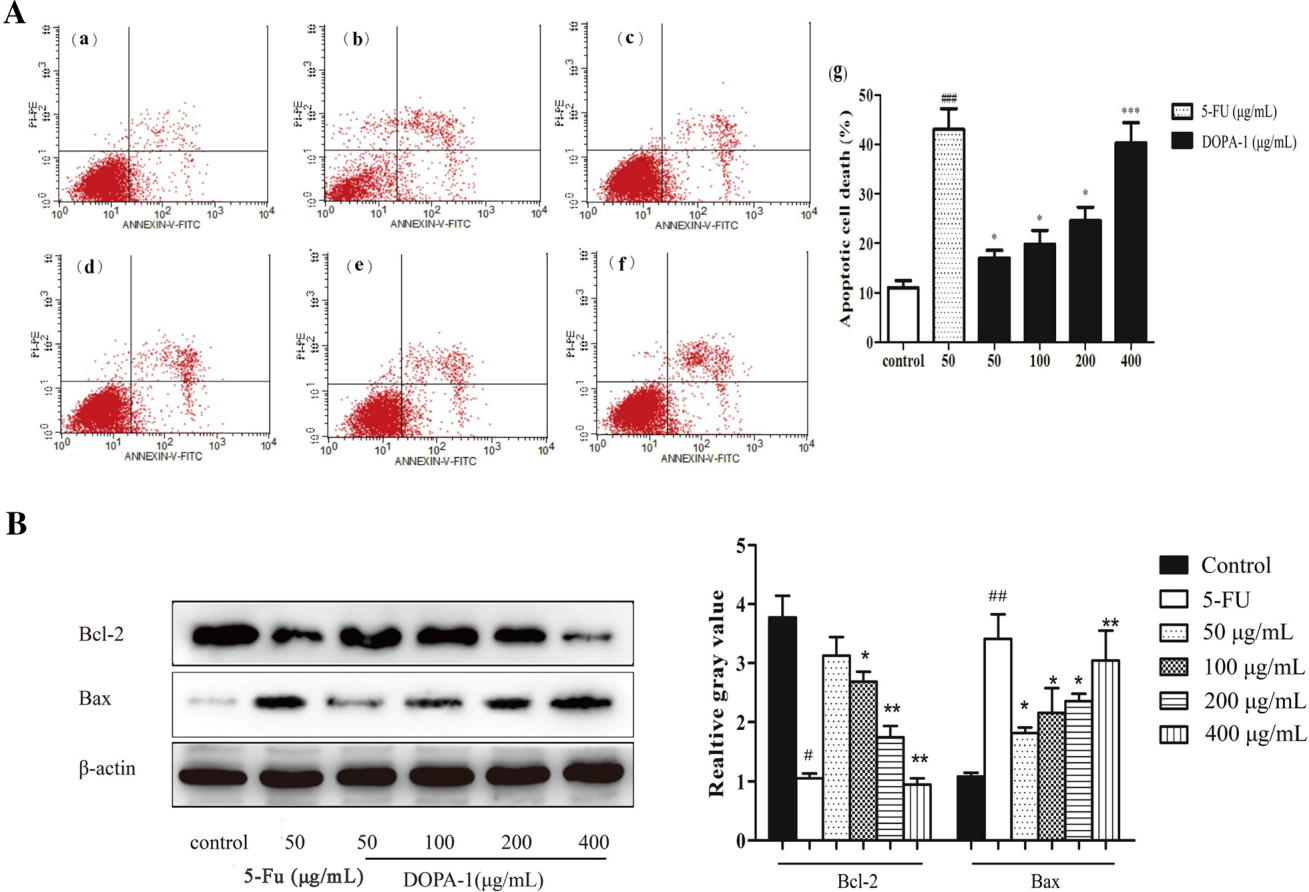
DOPA-1 treatment induces dose-dependent HepG-2 cell apoptosis. **A** Flow cytometric analysis of HepG-2 cell apoptosis with Annexin V-FITC and PI labeling. Subpanels: (a) untreated control group, (b) 50 μg/mL 5-FU, (c) 50 μg/mL DOPA-1, (d) 100 μg/mL DOPA-1, (e) 200 μg/mL DOPA-1, (f) 400 μg/mL DOPA-1, and (g) the calculated apoptotic rate for each treatment group. **B** Western blotting analysis and quantitation of Bax and Bcl-2 expression. Data are presented as the mean ± SD of three independent experiments. *p < 0.05, **p < 0.01, ***p < 0.001, ^#^*p* < 0.05, ^##^*p* < 0.01, compared to the blank control group

In order to examine the rate of apoptosis, Annexin VFITC/PI double staining was used in association with flow cytometry. As shown in Fig. 8A, the rate of apoptosis observed in cultures treated with 50, 100, 200, or 400 μg/mL DOPA-1 was 16.98, 19.82, 24.58, and 40.29%, respectively. Thus, similar to our earlier data, apoptosis increased in a dose-dependent manner and was significantly higher in the DOPA-1-treated groups than in the control group (11.0%).

### Effects of DOPA-1 on apoptosis-related protein expression

In this analysis, we focused on the protein expression of both anti- and pro-apoptotic factors in DOPA-1-treated HepG-2 cells. The expression of the anti-apoptotic protein Bcl-2 gradually decreased with increasing DOPA-1 concentration, while expression of the pro-apoptotic protein Bax gradually increased (Fig. 8B). This Bax/Bcl-2 ratio is known to be an important factor in regulating apoptosis. Following treatment with 50, 100, 200, or 400 μg/mL of DOPA-1 for 24 h, the Bax/Bcl-2 ratio gradually increased and the excess Bax formed homodimers, leading to cell apoptosis. While the full underlying mechanism still requires additional investigation, these data imply that DOPA-1-induced HepG-2 cell apoptosis is likely regulated by the expression of apoptosis-related proteins including Bax and Bcl-2.

## Discussion

Polysaccharides are carbohydrate chains of monosaccharides that are connected through glycosidic bonds. It is widely accepted that polysaccharide activity varies depending on its structure [23]. Thus, the type of glycosidic bonds in the polysaccharide backbone is a key factor in determining the functional effects of polysaccharides. For example, the presence of β-(1 → 3) linkages in the main chain of the glucan and additional β-(1 → 6) branch points have been shown to be necessary for anti-tumor activity [24]. Indeed, a previous study showed that the polysaccharide fractions of *Agaricus blazei* fruiting bodies have high anti-tumor activity and that their main glucan linkages are β-(1 → 6) and β-(1 → 3) [25]. In the present study, DOPA-1 appears to contain (1 → 3), (1 → 2), and (1 → 6) linkages in the main or branch chains, suggesting that it may account for the observed anti-tumor activity.

Previous studies have shown that elevated ROS levels may be involved in anti-tumor drug-induced cell death [26]. ROS are a group of oxygenated compounds produced during exogenous oxidation and intracellular aerobic metabolism [27]. Oxidative stress occurs when the rate at which ROS are cleared by the intracellular anti-oxidant system is slower than the rate at which they are produced [28]. High concentrations of ROS are known to upregulate proapoptotic protein expression, regulate the conformation of adenine nucleotide translocator protein in the inner mitochondrial membrane, and induce mitochondrial membrane permeability. ROS-mediated oxidative stress also reduces MMP, further activating cytochrome c and other apoptosis-inducing factors. As the mitochondrial membrane becomes more permeable, intracellular ROS will continue to rise, until tumor cell apoptosis is induced [29, 30]. Our results showed that DOPA-1 treatment increases intracellular ROS levels, as well as mitochondrial membrane permeability. Treatment also decreased MMP. These findings suggest that ROS and changes in the mitochondrial membrane play a significant role in the mechanism of DOPA-1 during cancer cell apoptosis.

In addition to ROS and mitochondrial function, we also evaluated the effects of DOPA-1 treatment on apoptosis-related protein expression. As a major modulator of apoptosis, the Bcl-2 protein family controls the release of mitochondrial apoptosis-inducing factors [31], which can be divided into pro-apoptotic proteins (such as Bax, Bak, and Bid) and anti-apoptotic proteins (such as Bcl-2, Bcl-xL, and Bcl-w) [32]. In this study, we found that DOPA-1 decreases Bcl-2 expression, while increasing Bax expression in HepG-2 cells. Thus, while the full mechanism underlying the anti-tumor effects of DOPA-1 still needs to be evaluated, these data suggest that this polysaccharide promotes HepG-2 cell apoptosis by regulating the expression of apoptosis-related proteins.

Notably, a polysaccharide recently isolated from *D. officinale* showed immune-modulating activity [33], and a novel heteroxylan isolated from the same plant had potential on anti-angiogenesis activities [34]. Those polysaccharides had different molecular weights and compositions compared to DOPA-1. It would be of great interest to further investigate if the three polysaccharides from the same plant have synergistic effects on anti-tumor activities.

## Conclusions

In this present study, we isolated, purified, and characterized a water-soluble polysaccharide from *D. officinale* grown in the Huoshan area, DOPA-1. To the best of our knowledge, this is the first time DOPA-1 has been evaluated with regards to its composition and anti-tumor mechanisms. This neutral homogeneous polysaccharide has an average molecular weight of 2.29 × 10^5^ Da and appears to be composed of mannose, glucose, and galactose at a molar ratio of 1:0.42:0.27. DOPA-1 inhibits in vitro tumor cell growth in a dose-dependent manner. The DOPA-1-mediated mechanism underlying tumor cell death involves ROS-induced mitochondrial dysfunction, namely increased membrane permeability and decreased MMP. Furthermore, DOPA-1-induced apoptosis also appears to be related to the downregulation of Bcl-2 protein expression and upregulation of Bax protein expression. In summary, this study provides insight into the composition and structure of the polysaccharides of *D. officinale* grown in the Huoshan area and highlights their distinct anti-tumor effects. This potent bioactive component could, therefore, potentially be further developed and used as an anti-tumor adjuvant drug.

## Abbreviations

DOPA-1: *D. officinale* polysaccharide
FBS: fetal bovine serum
MTT: 3-(4,5-dimethylthiazol-2-yl)-2,5-diphenyltetrazolium bromide
DMEM: Dulbecco’s modified Eagle’s medium
5-FU: 5-fluorouracil
PI: propidium iodide
DMSO: dimethyl sulfoxide
ROS: reactive oxygen species
HPLC: high performance liquid chromatography
RID: refractive index detector
FT-IR: Fourier transform infrared
NMR: nuclear magnetic resonance
TFA: trifluoroacetic acid
GC: gas chromatography
PBS: phosphate buffered saline
MMP: mitochondrial membrane potential
SDS: sodium dodecyl sulfate
PVDF: polyvinylidene fluoride
BSA: bovine serum albumin
PBST: PBS + Tween 20
SD: standard deviation
ANOVA: analysis of variance
